# Retraction: Conversion of human umbilical cord mesenchymal stem cells in Wharton’s jelly to dopamine neurons mediated by the Lmx1a and Neurturin *In Vitro*: Potential therapeutic application for Parkinson’s disease in a rhesus monkey model

**DOI:** 10.1371/journal.pone.0242032

**Published:** 2020-11-02

**Authors:** The PLOS ONE Editors

Following the publication of this article [1], concerns were raised regarding the results presented in Figs 9, 10, 14C, and 14J. Specifically,

- Several repetitive elements have been detected in the background of Fig 9, affecting all lanes. In addition, unusually straight horizontal and vertical discrepancies appear to cut the signal of each band presented in this figure. The original western blot data provided by the corresponding author did not match the figure as presented in the article and did not explain the appearance of the repetitive elements and other discrepancies observed in the figure.
- In Fig 10, the background between the top of the blot and the 43 kDa marker in lane 1 appears similar to the background of the same area in lane 2, lane 3 and lane 4, and the background between 34 kDa marker and the bottom of the blot in lane 1 appears similar to the background of the same area in lane 2, lane 3, and lane 4. In addition, there appear to be unusually straight horizontal and vertical lines surrounding the band presented in lane 1. The original western blot data provided by the corresponding author did not match the figure as presented in the article and did not explain the similarities observed in the background of the separate lanes and other discrepancies observed in the figure.
- Black rectangular squares have been detected in Fig 14C and Fig 14J that obscure the signal present in the underlying areas of these panels. The corresponding author provide underlying data for these panels, however the data provided for Fig 14C did not match the published panel, and the data provided for Fig 14J only partially matched the published panel not including the region of concern.

In light of the concerns affecting multiple figure panels that question the integrity of these data, the *PLOS ONE* Editors retract this article.

MY, SoL, and HL did not agree with the retraction. MS, YZ, WaW, ZH, DT, ShL, XW, and WeW either did not respond or could not be reached.
